# The feasibility of ^18^F-AlF-NOTA-PRGD2 PET/CT for monitoring early response of Endostar antiangiogenic therapy in human nasopharyngeal carcinoma xenograft model compared with ^18^F-FDG

**DOI:** 10.18632/oncotarget.8402

**Published:** 2016-03-26

**Authors:** Yanfen Cui, Huanhuan Liu, Sheng Liang, Caiyuan Zhang, Weiwei Cheng, Wangxi Hai, Bing Yin, Dengbin Wang

**Affiliations:** ^1^ Department of Radiology, Xinhua Hospital, Shanghai Jiao Tong University School of Medicine, Shanghai 200092, China; ^2^ Department of Nuclear Medicine, Xinhua Hospital, Shanghai Jiao Tong University School of Medicine, Shanghai 200092, China; ^3^ School of Pharmacy, Shanghai Jiao Tong University, Shanghai 200240, China; ^4^ Med-X Research Institute, Shanghai Jiao Tong University, Shanghai 200030, China; ^5^ Med-X Ruijin Hospital Micro PET/CT Research Center, Ruijin Hospital, Shanghai Jiao Tong University School of Medicine, Shanghai 200025, China

**Keywords:** antiangiogenic therapy, Endostar, ^18^F-FDG, ^18^F-AlF-NOTA-PRGD2, NPC

## Abstract

**Purpose:**

Radiolabeled arginine-glycine-aspartic acid (RGD) peptides have been developed for PET imaging of integrin avβ3 in the tumor vasculature, leading to great potential for noninvasively evaluating tumor angiogenesis and monitoring antiangiogenic treatment. The aim of this study was to investigate a novel one-step labeled integrin-targeted tracer, ^18^F-AlF-NOTA-PRGD2, for PET/CT for detecting tumor angiogenesis and monitoring the early therapeutic efficacy of antiangiogenic agent Endostar in human nasopharyngeal carcinoma (NPC) xenograft model.

**Experimental design and results:**

Mice bearing NPC underwent ^18^F-AlF-NOTA-PRGD2 PET/CT at baseline and after 2, 4, 7, and 14 days of consecutive treatment with Endostar or PBS, compared with ^18^F-FDG PET/CT. Tumors were harvested at all imaging time points for histopathological analysis with H & E and microvessel density (MVD) and integrin avβ3 immunostaining. The maximum percent injected dose per gram of body weight (%ID/gmax) tumor uptake of ^18^F-AlF-NOTA-PRGD2 PET/CT was significantly lower than that in the control group starting from day 2 (*p* < 0.01), much earlier and more accurately than that of ^18^F-FDG PET/CT. Moreover, a moderate linear correlation was observed between tumor MVD and the corresponding tumor uptake of ^18^F-AlF-NOTA-PRGD2 PET/CT (*r* = 0.853, *p* < 0.01).

**Conclusions:**

^18^F-AlF-NOTA-PRGD2 PET/CT can be used for *in vivo* angiogenesis imaging and monitoring early response to Endostar antiangiogenic treatment in NPC xenograft model, favoring its potential clinical translation.

## INTRODUCTION

Angiogenesis, the formation of new blood vessels, has long been recognized to play a critical role in tumor growth, invasion, and metastasis [1, 2]. Targeting of tumor angiogenesis pathway has been exploited as an effective therapeutic approach for cancer treatment [3–5]. Nasopharyngeal carcinoma (NPC), one of the most common malignancies of the head and neck in China and Southeast Asia, is highly vascularized. Moreover, some studies have investigated the potential of antiangiogenic agents alone or in combination with chemoradiotherapy in advanced stages of NPC with encouraging results [6–9]. However, not all patients would benefit from a specific antiangiogenic therapy and some patients may not respond [10, 11]. It is therefore in great demand to develop an alternative approach to identify patients who most likely benefit from antiangiogenic treatment, to detect emerging resistance, and to monitor early therapeutic efficacy [12].

Histopathologic evaluation of microvessel density (MVD), a prognostic indicator of progression, is not practical for routinely evaluating tumor angiogenesis due to its invasive nature of the procedure [13]. Noninvasive imaging technologies such as dynamic contrast-enhanced (DCE) magnetic resonance imaging (MRI) or computed tomography (CT), used to provide evidence on tumor blood flow, permeability, and volume, are technically challenging and cannot directly and effectively quantify the changes of post-treatment tumoral vascularity [14–16]. Positron emission tomography (PET) using ^18^F-FDG (2-deoxy-2-18F-fluoro-D-glucose) also was used to monitor antiangiogenic therapy by determining glucose metabolism changes, but it may not be an ideal modality because it is not a tumor-specific radiotracer. Therefore, molecular imaging targeting specific pathways involved in angiogenesis is warranted for specific monitoring of some molecular changes as early therapeutic effects via antiangiogenesis, with the benefit that it allows repetitive noninvasive follow-ups during the course of therapy.

Integrin avβ3, an adhesion molecule responsible for the regulation of tumor angiogenesis and invasion, is highly expressed on activated and proliferating endothelial cells during tumor angiogenesis and several types of cancer cells, but not in quiescent blood vessels [17–19]. Moreover, peptides containing arginine-glycine-aspartic acid (RGD) can specifically and strongly bind to integrin avβ3, therefore, a series of radiolabeled RGD peptide probes have been developed in a bid to visualize and quantify integrin αvβ3 expression *in vivo* and some of them are undergoing clinical trials [20–22]. However, only a few of studies have been performed on the use of RGD tracers to evaluate the therapeutic efficacy of antiangiogenic agents, especially in the territory of human NPC [23–26].

Endostar, a novel modified recombinant human endostatin, approved by the China Food and Drug Administration (CFDA) for the treatment of non-small cell lung cancer (NSCLC) in 2005, has showed strong antiangiogenic effect on a variety of xenotrans planted tumors and cancer patients, including NPC [27–32]. In the present study, we evaluated the potential of ^18^F-AlF-NOTA-PRGD2, a novel one-step labeled integrin-targeted tracer, for PET/CT imaging to detect tumor angiogenesis in human NPC xenograft model. In addition, we investigated its feasibility to monitor the early response to Endostar antiangiogenic therapy, with the comparison of ^18^F-FDG.

## RESULTS

### Chemistry and radiochemistry

The labeling efficiency for ^18^F-AlF-NOTA-PPRGD2 varied from 5% to 25% depending on the reaction volumes. The total synthesis time was about 30 min without HPLC purification. The radiochemical purity was over 97%.

### Integrin αvβ3 expression validation

**Figure 1:**
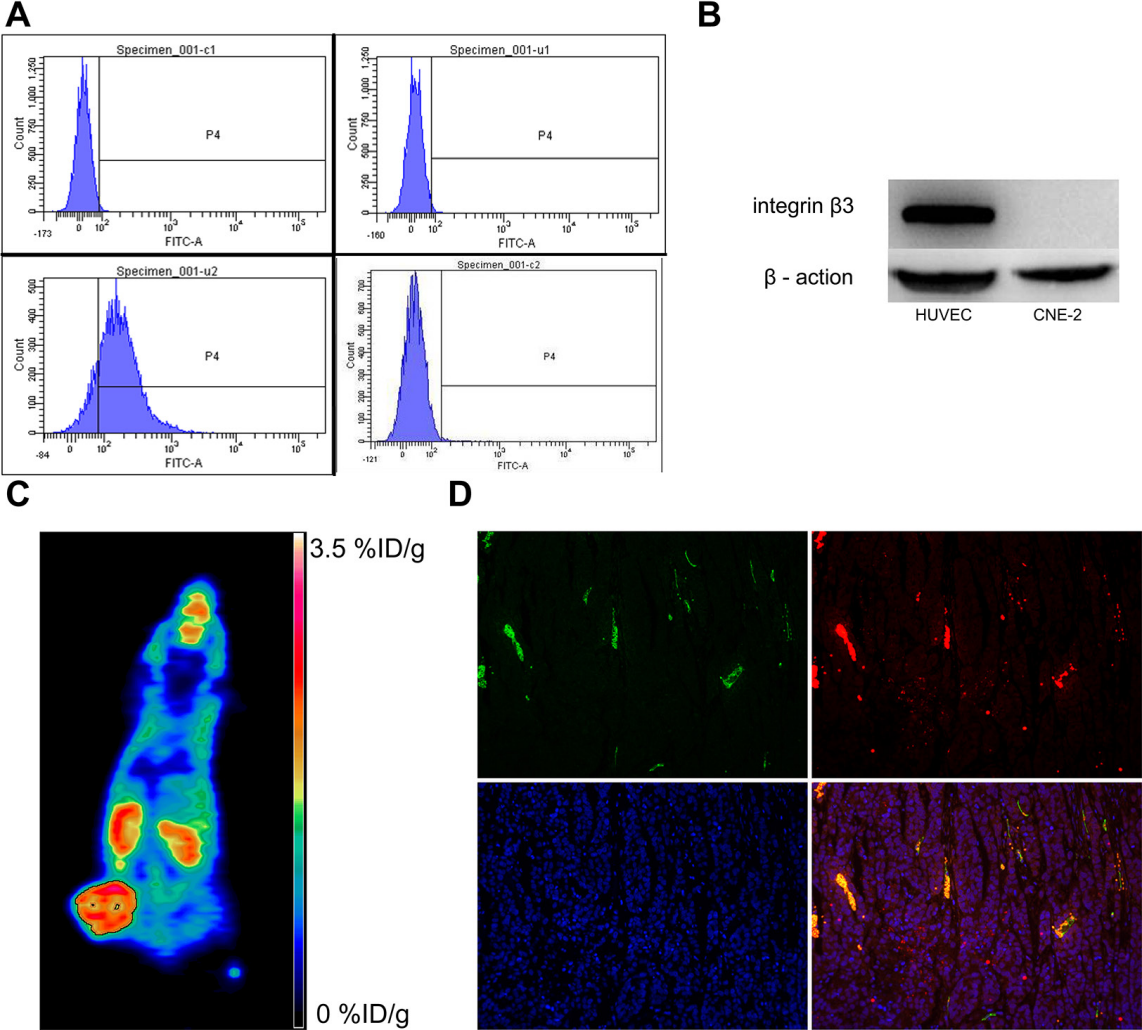
Analysis of integrin αvβ3 expression in CNE-2 cells and tumor sections (**A**) Flow cytometric analysis and (**B**) Western blot studies confirmed that CNE-2 cells does not express integrin αvβ3. (**C**) MicroPET imaging of CNE-2 bearing mice at 1 h after intravenous injection of ^18^F-AIF-NOTA-PRGD2, and (**D**) *Ex vivo* CNE-2 tumor tissue immunofluorescence staining of co-staining of CD31(green) and CD61(integrin β3, red) and DAPI.

As shown in Figure 1A, the FACS analysis demonstrated that positive rate of CNE-2 cells stained with anti-human integrin αvβ3 monoclonal antibody was only 0.7%, with 84.4% for HUVECs as compared. The Western blot study confirmed the FACS findings (Figure 1B), indicating that the integrin β3 expression on the CNE-2 cell was negligible. The representative micro PET/CT imaging of CNE-2 bearing mice 1 h after injection of ^18^F-AIF-NOTA-PRGD2 was shown in Figure 1C. The tumor could be clearly visualized with excellent contrast to contralateral background. Moreover, high radioactivity accumulation of the tumor was detected.

For the validation of intergrin αvβ3 expression on CNE-2 tumor tissues, we should stained tumor sections with both anti-human and anti-murine integrin αvβ3 antibodies, because the CNE-2 tumor cells are of human origin and the tumor vasculature endothelial cells are of mouse origin [33]. On one hand, we have validated that there is no integrin αvβ3 expression on CNE-2 cell *in vitro* as above. On the other hand, most of β3 was found to bind to αv (αvβ3) or αIIb (αIIbβ3), but in tumors the latter was hardly expressed. Hence, we only performed immunofluorescence against murine β3 to confirm the integrin αvβ3 expression on the tumor vasculature endothelial cells in CNE-2 tumor tissue. As shown in Figure 1D, the CNE-2 tumor tissues were highly vascularized indicated by strong CD31 staining. Moreover, the co-localization of integrin β3 and CD31 indicated that the integrin β3 expression in the CNE-2 tumor tissues was mainly derived from the tumor blood vessels.

### Effects of Endostar on CNE-2 tumor growth

**Figure 2:**
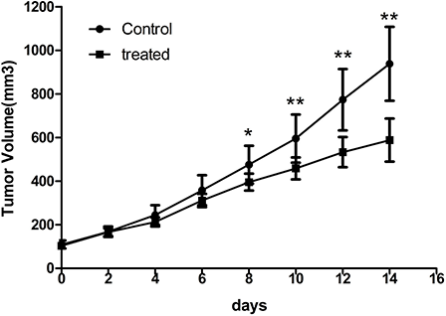
Endostar treatment inhibits growth of CNE-2 tumors Comparison of tumor volumes in the control and Endostar-treated group in established CNE-2 xenografts. Tumor volume was determined by caliper measurements. Error bars denote standard errors. (*p < 0.05; **p < 0.01).

The effective antiangiogenic therapy with Endostar was carried out in CNE-2 tumor-bearing mice. Daily administration of Endostar consecutively for 14 days tended to slow the tumor growth. As displayed in Figure 2, a much less time-dependent increase in tumor growth was observed in the treated group than the control. No early resistance or tumor progression was found in our study. In addition, the tumor volumes in the treated group were significantly reduced compared with those in the control group starting from day 8 (*P* < 0.05). At the end of the study, the tumor volumes reached 937.97 ± 91.32 mm^3^ for the treatment group versus 588.32 ± 70.89 mm^3^ for the control group (*P* < 0.01). No observable body weight loss or any other side effects were observed during the treatment period, indicating that the dosage was safe.

### Monitoring the antiangiogenic effect of Endostar by microPET/CT

**Figure 3:**
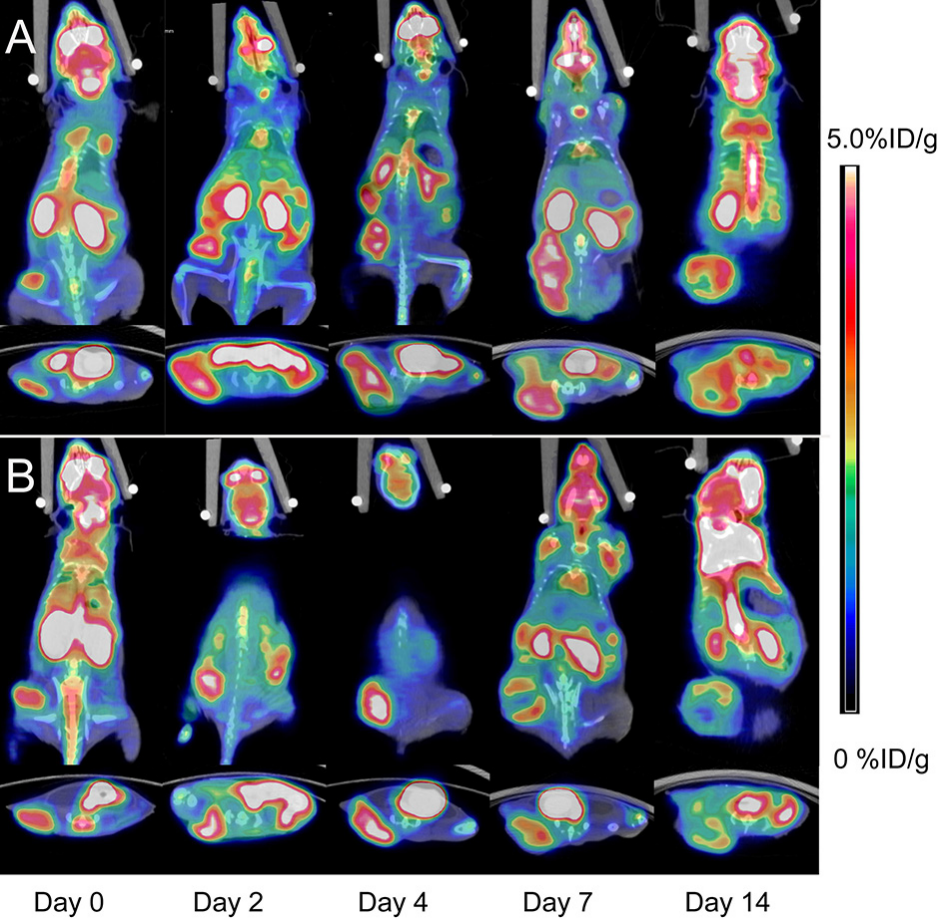
Representative microPET/CT images of mice bearing CNE-2 NPC tumors at 1 h after intravenous injection of ^18^F-FDG on days 0, 2, 4, 7, and 14 after treatments was initiated (**A**) Saline control. (**B**) Endostar treatment.

**Figure 4:**
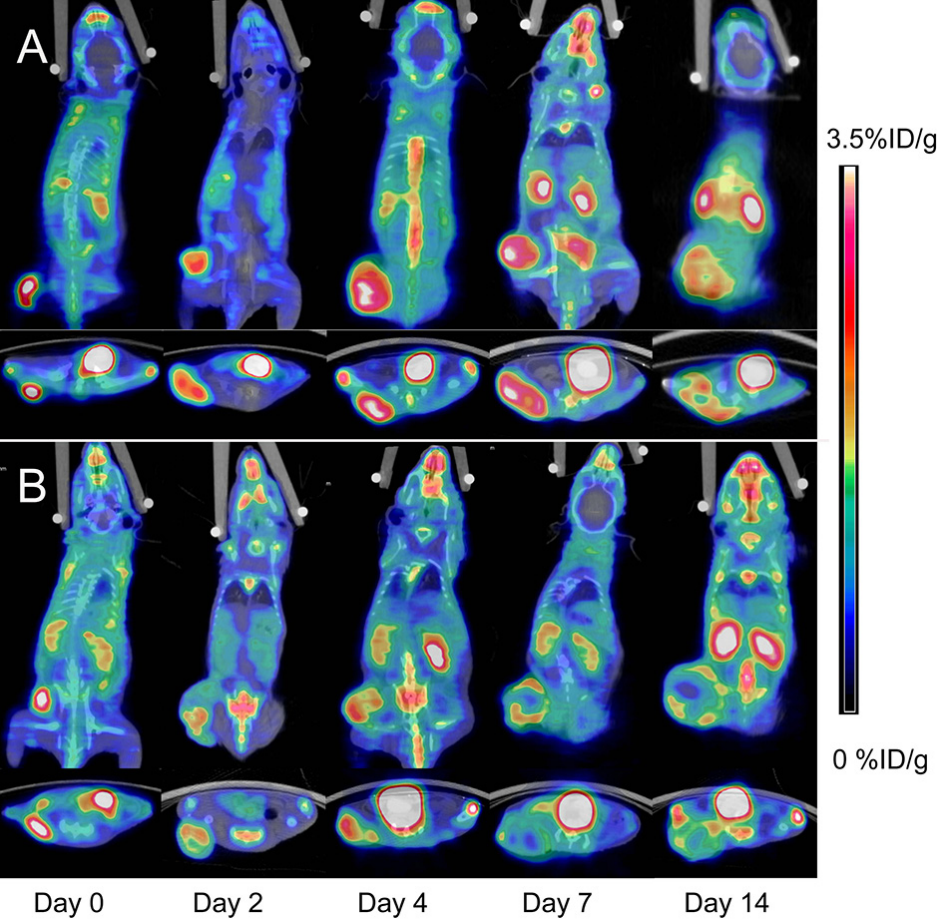
Representative microPET/CT images of mice bearing CNE-2 NPC tumors at 1 h after intravenous injection of ^18^F-AIF-NOTA-PRGD2 on days 0, 2, 4, 7, and 14 after treatments was initiated (**A**) Saline control. (**B**) Endostar treatment.

The static ^18^F-FDG and ^18^F-AIF-NOTA-PRGD2 PET/CT imaging at baseline and days 2, 4, 7, and 14 post-treatment were shown in Figure 3 and Figure 4, respectively. Visually, the radioactivity was obviously lower in the tumors of treatment group than those in the control mice, especially for the group with ^18^F-AIF-NOTA-PRGD2. Furthermore, the radioactivity distribution in the treatment group become more and more heterogeneous due to tumor necrosis, while the radioactivity accumulated in the control tumors was relatively homogeneous.

**Figure 5:**
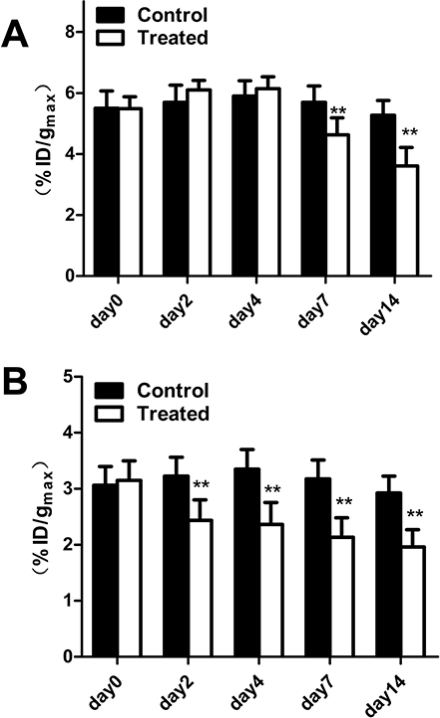
Quantitative microPET/CT ROI analysis of tumor uptake (**A**) Decreased tumor uptake of ^18^F-FDG was observed on days 7. (**B**) Decreased tumor uptake of ^18^F-AIF-NOTA-PRGD2 was observed on days 2. (*p < 0.05; **p < 0.01).

Tumor uptake (%ID/g_max_) calculated from PET/CT images are presented in Figure 5. There was little fluctuation of ^18^F-FDG or ^18^F-AIF-NOTA-PRGD2 uptake in the control mice at different time point. The tumor uptake values (%ID/g_max_) of ^18^F-FDG in control group were 5.50 ± 0.57, 5.70 ± 0.56, 5.90 ± 0.50, 5.70 ± 0.53, and 5.28 ± 0.48, while these values in the treatment group were 5.49 ± 0.39, 6.10 ± 0.32, 6.14 ± 0.39, 4.63 ± 0.57, and 3.61 ± 0.60 on days 0, 2, 4, 7, and 14 post-treatment, respectively (Figure 5A). There is no significant difference of ^18^F-FDG uptake in Endostar treated tumors until day 7 (*p* < 0.01) compared with the control group. For the ^18^F-AIF-NOTA-PRGD2 PET/CT imaging, the tumor uptake values (%ID/g_max_) in the treatment group were 3.15 ± 0.35, 2.44 ± 0.37, 2.36 ± 0.39, 2.14 ± 0.35, and 1.96 ± 0.31, while the values in the control group were 3.06 ± 0.33, 3.23 ± 0.34, 3.35 ± 0.35, 3.18 ± 0.34 and 2.93 ± 0.30 on days 0, 2, 4, 7, and 14 post-treatment, respectively (Figure 5B). Compared to ^18^F-FDG microPET/CT imaging, tumor uptake values of ^18^F-AIF-NOTA-PRGD2 in the treatment group were significantly lower than those of the control group starting from day 2 (*p* < 0.01), indicating that the tumor growth inhibition could be reflected as early as 2 days after Endostar treatment with ^18^F-AIF-NOTA-PRGD2 PET/CT imaging, much earlier than ^18^F-FDG PET/CT imaging.

### *Ex vivo* tumor tissue analyses for assessing response to antiangiogenic therapy

**Figure 6:**
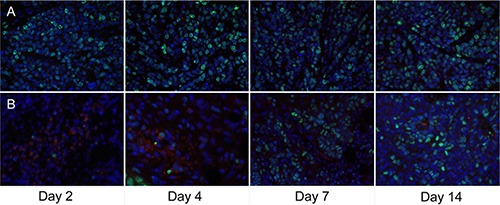
Immunofluorescence staining of tumor vasculature and murine integrin αvβ3 expression with antibodies against CD31 (green) and CD61 (integrin β3, red) in the Endostar treatment group at different time point

The immunofluorescence staining of CD31 and CD61 in *ex vivo* CNE-2 tumor tissues in the treated group at baseline and 2, 4, 7, and 14 days after Endostar treatment were evaluated. As displayed in Figure 6, the tumor angiogenesis was found significantly inhibited as early as day 2 post-treatment, compared with that of pre-treatment (day 0), and the inhibition lasted to the end of the 2-week treatment. Interestingly, the expression of integrin αvβ3 on the CNE-2 tumor cell was negligible, and almost all the murine β3 was co-localized with the CD31, all these results demonstrated that the integrin αvβ3 was solely expressed on the tumor vasculature in the CNE-2 tumor tissue.

**Figure 7:**
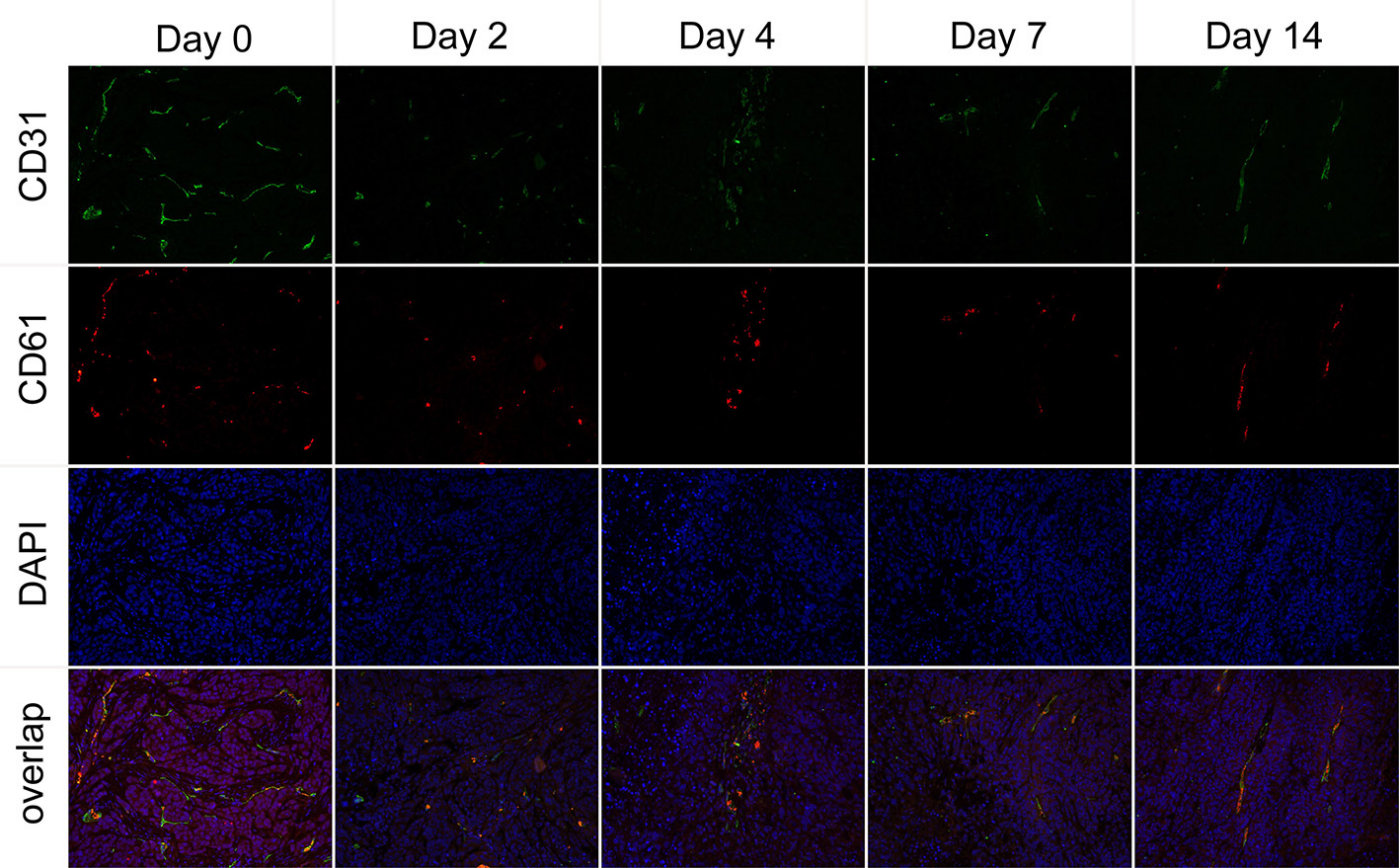
*Ex vivo* overlay staining of Ki-67 (green) and F4/80 (red) of CNE-2 tumors on days 2, 4, 7, and 14 (**A**) The control group. (**B**) Endostar treatment group.

For the macrophage-specific marker F4/80 (Figure 7), a slightly increased macrophage infiltration indicating inflammatory reaction was observed at the early stage of Endostar treatment, whereas it began to decrease from day 7 after treatment, compared with control tumors, contrary to the trend of tumor cell proliferation Ki-67.

**Figure 8:**
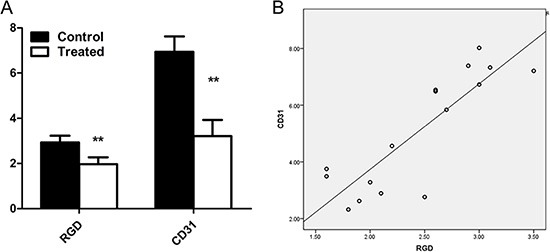
Comparison and correlation analysis of ^18^F-AIF-NOTA-PRGD2 tumor uptake and MVD (**A**) Comparison of tumor uptake of ^18^F-AIF-NOTA-PRGD2 and the corresponding MVD at the end of treatment in the control and Endostar-treated group. (**B**) Moderate correlation between tumor uptake of ^18^F-AIF-NOTA-PRGD2 at the end of therapy and corresponding MVD with *r*^2^ value of 0.728 were obtained. (**p < 0.01).

Furthermore, the correlations between ^18^F-AIF-NOTA-PRGD2 tumor uptake at the end of therapy and the corresponding MVD are shown in Figure 8. A positive and significant linear relationships with *r*^2^ = 0.728 was observed between them. The MVD was significantly lower in the Endostar-treated tumors than that in the control tumors (3.21 ± 0.72% vs. 6.94 ± 0.68%; *P* < 0.001), consistent with the tendency of the corresponding tumor uptake.

## DISCUSSION

In our present study, we demonstrated that ^18^F-AIF-NOTA-PRGD2 enable the visualization and quantification of tumor angiogenesis in the CNE-2 NPC xenograft model, for which intergrin αvβ3 was solely expressed on the tumor vasculature, similar to most kinds of HNSCC cell line and cancer patients [22, 34]. Moreover, ^18^F-AIF-NOTA-PRGD2 could effectively monitor the early response to the antiangiogenic therapy of Endostar in the CNE-2 NPC xenograft model.

Endostar, as an antiangiogenic agent, on one hand, can cause cytostatic effects rather than cytotoxic effects, that is, antiangiogenic treatment could lead to a delay or stop of tumor progression rather than tumor shrinkage. With a therapy regimen of consecutive daily injection for 14 days in our study, Endostar impeded CNE-2 tumor growth without evident tumor regression and a significant tumor growth inhibition was not observed until day 8. On the other hand, Endostar can inhibit the formation of tumor newborn vascular endothelial cells, resulting in the decrease of MVD, which led to the decrease of integrin expression levels and corresponding targeted imaging probe uptake in tumors. In our study, changes in the tumor uptake of ^18^F-AIF-NOTA-PRGD2 were observed as early as day 2 after the initiation of Endostar treatment, before any significant volumetric changes of tumors. Moreover, the reduction in tumor accumulation of ^18^F-AIF-NOTA-PRGD2 was much earlier than ^18^F-FDG detectable tumor metabolic changes on day 7 post-treatment.

^18^F-FDG is the most commonly used PET imaging agent and has been widely applied in the clinical context for tumor diagnosis, cancer staging, and therapeutic effect evaluation. However, ^18^F-FDG is not a tumor-specific radiotracer and may shows complex changes when used to monitor antiangiogenic therapy. It is interesting to note that, ^18^F-FDG uptake was somewhat increased on day 2 and 4 compared with that in the control group, possibly because of the combined effect of slightly increased macrophage infiltration (F4/80 staining) and reduced tumor cell proliferation (Ki-67 index) at the early stage of Endostar-treated tumors. It has been well documented that ^18^F-FDG can lead to a false-positive in forms of inflammation, infection, and granulomatous disease [35, 36], which may be the limitation of ^18^F-FDG in the determination of therapeutic response.

Thus far, an increasing number of RGD-based pepetides carrying a range of radionyclides (e.g., ^18^F, ^99m^Tc, ^89^Zr, ^68^Ga, ^111^In, and ^64^Cu) have been developed using various strategies for radiolabeling techniques and peptide modification for integrin αvβ3 expression. With the well-suited physical half-life of 109.7 min, ^18^F is the most popular radioisotope for routine clinical use. Consequently, several ^18^F-labeled RGD peptide tracers, including ^18^F-galacto-RGD [34], ^18^F-AH11585 [21], have been tested in oncologic patients. Futhermore, ^18^F-FPPRGD2 [37] has been approved by the Food and Drug Administration (FDA). Many attempts, including the cyclization of the peptides, the synthesis of multimeric RGD peptides and PEGlaytion, have been made to pursue more ideal RGD probes with high tumor targeting capability [38]. However, the monomeric cyclic RGD and time-consuming multiple-step synthetic procedure and relatively low yield had hindered their widespread use [39]. Therefore, a novel one-step labeled tracer ^18^F-AlF-NOTA-PRGD2 has been prepared successfully by direct labeling ^18^F-aluminum-fluoride complex with a pre-attached chelator on the dimeric RGD peptides with the application of chelation chemistry, without the need of HPLC [40, 41]. In our study, we proved that ^18^F-AlF-NOTA-PRGD2 enabled visualization of tumor angiogenesis by targeting integrin avβ3 in CNE-2 xenograft model with good imaging quality.

Additionally, we investigated the potential of ^18^F-AlF-NOTA-PRGD2 to accurately assess the early antiangiogenic efficacy of Endostar. Our longitudinal imaging results indicated that even though much higher tumor uptake was found in ^18^F-FDG PET/CT imaging, therapeutic effect was more clearly and much earlier reflected by ^18^F-AlF-NOTA-PRGD2 PET/CT imaging. Furthermore, the tumor uptake showed significantly changes in the Endostar-treated tumors after two-week treatment than those in the control tumors, consistent with the tendency of the corresponding MVD. More importantly, a positive and significant correlation was found between the tumor MVD and the corresponding^18^F-AlF-NOTA-PRGD2 tumor uptake. The co-localization of CD31 and CD61 support these results, which strongly demonstrate that the antiangiogenic effects of Endostar can be monitored by quantitative ^18^F-AlF-NOTA-PRGD2 PET/CT imaging.

Our results are in agreement with those of recently published studies about the use of radiolabeled RGD peptides to monitor the antiangiogenic treatment efficacy in animal models. For example, Battle et al. [26]. demonstrated that the tumor uptake of ^18^F-AH111585 decreased significantly from day 2 onward during the 14-days treatment of sunitinib, a small-molecular tyrosine kinase inhibitor (TKI), in integrin αvβ3-positive U87MG tumors models, in line with the corresponding MVD reduction. Similarly, ZD4190, another TKI, appeared to exert a significant decrease of ^18^F-AH111585 uptake in Calu-6 tumors [42] as well as ^18^F-FPPRGD2 uptake in MDA-MB-435 breast tumors [25], although the integrin αvβ3 expression level on the latter cell surface was much higher than the former [42]. Collectively, all these results demonstrated that PET/CT with RGD-based probe trace was promising for the assessment of antiangiogenic therapy response.

However, we have to bear in mind that different antiangiogenic agents may have distinct effects on tumor integrin expression, since integrin αvβ3 is not only expressed on the tumor vascular endothelial cells, but also on some kinds of tumor cells [19]. For example, it has been reported that only murine integrin expression on tumor vasculature is down-regulated after treatment with low-dosage Abraxane, but that on tumor cells was almost unaffected [33], whereas the ZD4190 may suppress the integrin expression both on the tumor vasculature endothelial cells and tumor cells by primarily targeting vascular endothelial growth factor receptor (VEGFR) and epidermal growth factor receptor (EGFR) tyrosine kinase activity [25]. In our study, the intergrin αvβ3 was solely expressed on the tumor vasculature in the CNE-2 tumor model, making it possible that ^18^F-AlF-NOTA-PRGD2 can directly correlate the tumor uptake of the tumor with corresponding MVD as well as can effectively monitor the early response to antiangiogenic effect of Endostar in the NPC xenograft model. However, we have to bear in mind that tumor uptake of a given radiotracer are not solely dependent on receptor expression. Several factors, such as vascular permeability, vascular density and volume, may affect the specific uptake. Trace kinetic modeling has been validated valuable for separating specific and nonspecific binding and may allow more sensitive and detailed quantification than simple SUV analysis [43, 44]. Therefore, further investigations are warranted to eliminate the influence of tumor microenvironment on the pharmacokinetics of ^18^F-AlF-NOTA-PRGD2.

## CONCLUSIONS

In our present study, we proved that ^1^8F-AlF-NOTA-PRGD2 PET/CT enabled the clear visualization of tumor angiogenesis and could monitor the therapeutic efficacy of antiangiogenesis in the CNE-2 human NPC xenograft model, which was much earlier and more accurately than ^18^F-FDG metabolic imaging and the anatomical structure changes. Therefore, it may be a promising radiotracer used for monitoring the early response from antiangiogenic therapy on tumor and could potentially be translated into clinical practice.

## MATERIALS AND METHODS

### General materials

Unless otherwise specified, all reagents obtained commercially were of analytical grade and used without further purification. No-carrier-added ^18^F- fluoride was obtained from an in-house PET trace cyclotron (HM-10, Sumitomo Heavy Industries Ltd, Japan). ^18^F-FDG was purchased from the Nuclear Pharmacy of Cardinal Health and reconstituted with sterile saline. The peptides pegylated dimeric RGD peptide PEG_3_-E[c(RGDyK)]_2_ (denoted as PRGD2) and NOTA-PRGD2 were synthesized by Chinese Peptide Company (Hangzhou, China). The female BALB/c nude mice (6–8 weeks old, 18–22 g body weight) were purchased from Shanghai Experimental Animal Center (Shanghai, China). The Endostar was provided by Simcere Pharmaceutical Research Co., Ltd.

### Synthesis and quality control of ^18^F- AlF-NOTA-PRGD2

^18^F- AlF-NOTA-PRGD2 was synthesized following previously reported procedure [45]. Briefly, aluminum chloride in 0.2 M sodium acetate buffer at pH = 4 (3 uL, 2 mM) was added into 0.1 mL aqueous [18F] fluoride (0.37 GBq) in a 1.0 mL V-vial. The solution was then heated at 100°C for 10 min to form the aluminum–fluoride complex. After the vial was cooled, NOTA-PRGD2 in 0.2 M sodium acetate buffer at pH = 4 (6 uL, 2 mM) was added, and then the vial was heated at 100°C for another 10 min. At the end of the reaction, the mixture was directly loaded on the C18 cartridge without High Performance Liquid Chromatography (HPLC) purification and then eluted with 0.3 mL of 80% ethanol/water with 2% acetic acid. The ethanol solution was evaporated with an argon flow, and the final product was then formulated in PBS solution for *in vivo* study.

### Cell culture

Human umbilical vein endothelial cells (HUVECs), obtained from American Type Culture Collection (ATCC, Manassas, VA), were cultured in DMEM culture medium. A poorly differentiated NPC cell lines CNE-2 (FDCC, Fu Dan IBS Cell Center) were grown in RPMI 1640 culture medium. Both culture media were supplemented with 10% fetal bovine serum (Gibco, Paisley, UK) and 1% penicillin / streptomycin at 37°C in a humidified atmosphere containing 5% CO_2_. The expression of integrin αvβ3 on the surface of CNE-2 cells lines was confirmed using flow cytometry and Western blot analysis. HUVECs, supposed to express high amounts of integrin αvβ3, were simultaneously analyzed as the positive control.

### Fluorescence-activated cell sorting analysis

CNE-2 cells were harvested using trypsin and washed with PBS, and then a single cell suspension containing 1 × 10^6^ cells was incubated with monoclonal mouse antibodies against human integrin αvβ3 (1:40, R & D Systems, MN, USA) for 2 h at room temperature. After 3 washes, cells were incubated with FITC-Labled goat anti-mouse immunoglobulin G (1:50; BD Biosciences, CA, USA) for 30 minutes and were analyzed by a FACS Calibur flow cytometer (Becton-Dickinson, Rutherford, NJ, USA). Antimouse IgG was used as control. This protocol is also applied to HUVECs cells.

### Western blotting

CNE-2 and HUVECs cells were trypsinized and lysed in RIPA lysis buffer. Lysates were centrifuged at 12000 g for 10 minutes and protein concentration was determined by using an enhanced BCA protein assay kit (Pierce Biotechnology, Inc., Rockford, IL, USA). Fifty ug proteins were separated by using 8% SDS-PAGE and transferred to the polyvinylidene fluoride (PVDF) membrane. After that, the PVDF membrane was blocked with 5% nonfat milk blocking buffer, and then incubated overnight at 4°C with rabbit anti-human CD61 polyclonal primary antibody (1:200; BD Biosciences, San Jose, USA), followed by incubating with the secondary antibodies: donkey anti-rabbit-IRDye 680 (Li-COR Biosciences). β-actin was used as the loading control, and the bands were detected by using enhanced chemiluminescence (ECL) (Millipore, USA).

### Animal models

All the experimental protocols were performed in accordance with guidelines of Institutional Animal Care and Use Committee of Xinhua Hospital Affiliated to Shanghai Jiao Tong University School of Medicine. After a 1-week adaptation period, mice were subcutaneously injected with 2 × 10^6^ CNE-2 cells in 0.2 mL serum-free media into the right hind flank. Tumors were allowed to grow to 100 mm^3^ before treatment (approximately one week after implantation).

### Experimental design

The detailed experimental designs are shown in Table 1. Mice with CNE-2 human NPC tumors were allocated to two groups: the imaging group (*n* = 32) and the immunohistochemical (IHC) staining group (*n* = 20). Each major group was then divided into control group and Endostar treatment group. For the treated group, Endostar dissolved in 0.9% saline was injected intraperitoneally at the dose of 20 mg/kg/d for consecutive 14 days, while mice in the control group received 0.9% saline at the same dose. Mice in the imaging group underwent ^18^F-FDG or ^18^F-AIF-NOTA-PRGD2 PET/CT at baseline and after 2, 4, 7, 14 days of treatment with Endostar or PBS, while mice in the histopathologic group were sacrificed on the corresponding imaging time points for immunofluorescence analysis with 3 mice on each time point. To evaluate therapeutic response, the tumor volume was assessed with caliper measurement using the formula: V = 1/2 * ab^2^ (a, length; b, width). Body weight was measured every other day.

**Table 1:** Experimental design for longitudinal 18F-FDG and ^18^F-AIF-NOTA-PRGD2 imaging of Endostar treatment efficacy and *ex vivo* histopathology

		Day 0	Day 2	Day 4	Day 7	Day 14
^18^F-FDG	Control (*n* = 8)	#	#	#	#	#, +
	Endostar (*n* = 8)	#	#	#	#	#, +
^18^F-AIF-NOTA-PRGD2	Control (*n* = 8)	#	#	#	#	#, +
	Endostar (*n* = 8)	#	#	#	#	#, +
Histology	Control (*n* = 10)	+	+	+	+	+
	Endostar (*n* = 10)	+	+	+	+	+

# = small-animal PET/CT; + = tumor tissue sampling.

### Small-animal PET/CT imaging

Small-animal PET/CT imaging was performed by using an Inveon system (Siemens Preclinical Solutions, Knoxville, Tennessee, USA). Ten minute static PET scans were acquired at the time point of one hour after injection of 3.7 MBq (100 μCi) ^18^F-FDG or ^18^F-AIF-NOTA-PRGD2 via tail vein, followed by a 10 min micro-CT scan to obtain anatomic information. For the ^18^F-FDG scans, mice were fasted for 6 h before tracer injection. Animals were anesthetized with 1.5% isoflurane in oxygen at 2 L/min and kept warm with a temperature-controlled heating system during the entire imaging procedure. The images were reconstructed by a three-dimensional ordered subsets expectation maximum (3D OSEM) algorithm without attenuation or scattering correction and were then processed by using Siemens Inveon Research Workplace 3.0 (IRW 3.0). The 3D regions of interest (ROIs) were manually drawn over the tumor to obtain the maximum standardized uptake value (SUV_max_). Given a tissue density of 1 g/cm^3^, these values were then divided by the injected activity to obtain an image ROI-derived mean and maximum percent injected dose per gram of body weight (%ID/g_max_).

### Immunofluorescence staining

Frozen tumor tissue sections (5 μm) were fixed with acetone for 20 min and dried in the air for 30 min at room temperature. After blocking with 2% BSA for 30 min, slices were incubated with hamster anti-mouse CD61 (integrin β3) antibody (1:200; BD Biosciences, San Jose, CA, USA) and rat anti-mouse CD31 antibody (1:200; BD Biosciences, San Jose, CA, USA) and then visualized with FITC-conjugated goat anti-hamster and Cy3-conjugated goat anti-rat secondary antibody (1:200; Santa Cruz Biotechnology, CA, USA). For the staining of F4/80 and Ki-67, slices were incubated with rat anti-mouse F4/80 antibody (1:200; BD Biosciences, San Jose, CA, USA) and rabbit anti-human Ki-67 antibody (1:200; BD Biosciences, San Jose, CA, USA) separately, and then visualized with Cy3-conjugated goat anti-rat and FITC-conjugated goat anti-rabbit secondary antibody (1:400; Jackson ImmunoResearch Laboratories). After washing three times with PBS, the whole slides were mounted with mounting medium containing 4′-6-diamidino-2-phenylindole (DAPI). Fluorescence images were acquired with an epifluorescence microscope (Olympus, X81).

All quantitative analyses of the immunofluorescence staining, expressed by the percentage of positive area versus the area of the entire section, were assessed by using Image J software. Among them, the percentage of CD31-positive area was introduced to express the relative microvessel density (MVD) (%) of the tumor.

### Statistical analysis

Quantitative data were expressed as mean ± SD. Means were compared using one-way ANOVA and the Student's *t* test with GraphPad Prism 5 (GraphPad Software Inc., La Jolla, CA). Pearson correlation test was performed for correlations between tumor uptake of ^18^F-AlF-NOTA-PPRGD2 and the corresponding CD31 density at the end of follow-up. A *P* value < 0.05 was considered statistically significant.

## References

[1] Folkman J (2002). Role of angiogenesis in tumor growth and metastasis. Semin Oncol.

[2] Bergers G, Benjamin LE (2003). Tumorigenesis and the angiogenic switch. Nat Rev Cancer.

[3] Wahl O, Oswald M, Tretzel L, Herres E, Arend J, Efferth T (2011). Inhibition of tumor angiogenesis by antibodies, synthetic small molecules and natural products. Curr Med Chem.

[4] Burke PA, DeNardo SJ (2001). Antiangiogenic agents and their promising potential in combined therapy. Crit Rev Oncol Hematol.

[5] Ferrara N, Kerbel RS (2005). Angiogenesis as a therapeutic target. Nature.

[6] Yoo DS, Kirkpatrick JP, Craciunescu O, Broadwater G, Peterson BL, Carroll MD, Clough R, MacFall JR, Hoang J, Scher RL, Esclamado RM, Dunphy FR, Ready NE (2012). Prospective trial of synchronous bevacizumab, erlotinib, and concurrent chemoradiation in locally advanced head and neck cancer. Clin Cancer Res.

[7] Yang S, Wu J, Zuo Y, Tan L, Jia H, Yan H, Zhu X, Zeng M, Ma J, Huang W (2010). ZD6474, a small molecule tyrosine kinase inhibitor, potentiates the anti-tumor and anti-metastasis effects of radiation for human nasopharyngeal carcinoma. Curr Cancer Drug Targets.

[8] Cao S, Durrani FA, Toth K, Rustum YM, Seshadri M (2011). Bevacizumab enhances the therapeutic efficacy of Irinotecan against human head and neck squamous cell carcinoma xenografts. Oral Oncol.

[9] Heiduschka G, Lill C, Schneider S, Seemann R, Kornek G, Schmid R, Kotowski U, Thurnher D (2014). The effect of cilengitide in combination with irradiation and chemotherapy in head and neck squamous cell carcinoma cell lines. Strahlenther Onkol.

[10] Sennino B, McDonald DM (2012). Controlling escape from angiogenesis inhibitors. Nat Rev Cancer.

[11] Scott BJ, Quant EC, McNamara MB, Ryg PA, Batchelor TT, Wen PY (2010). Bevacizumab salvage therapy following progression in high-grade glioma patients treated with VEGF receptor tyrosine kinase inhibitors. Neuro Oncol.

[12] Sessa C, Guibal A, Del Conte G, Ruegg C (2008). Biomarkers of angiogenesis for the development of antiangiogenic therapies in oncology: tools or decorations?. Nat Clin Pract Oncol.

[13] Foote RL, Weidner N, Harris J, Hammond E, Lewis JE, Vuong T, Ang KK, Fu KK (2005). Evaluation of tumor angiogenesis measured with microvessel density (MVD) as a prognostic indicator in nasopharyngeal carcinoma: results of RTOG 9505. Int J Radiat Oncol Biol Phys.

[14] Morgan B, Thomas AL, Drevs J, Hennig J, Buchert M, Jivan A, Horsfield MA, Mross K, Ball HA, Lee L, Mietlowski W, Fuxuis S, Unger C (2003). Dynamic contrast-enhanced magnetic resonance imaging as a biomarker for the pharmacological response of PTK787/ZK 222584, an inhibitor of the vascular endothelial growth factor receptor tyrosine kinases, in patients with advanced colorectal cancer and liver metastases: results from two phase I studies. J Clin Oncol.

[15] Akisik MF, Sandrasegaran K, Bu G, Lin C, Hutchins GD, Chiorean EG (2010). Pancreatic cancer: utility of dynamic contrast-enhanced MR imaging in assessment of antiangiogenic therapy. Radiology.

[16] Bradley DP, Tessier JL, Checkley D, Kuribayashi H, Waterton JC, Kendrew J, Wedge SR (2008). Effects of AZD2171 and vandetanib (ZD6474, Zactima) on haemodynamic variables in an SW620 human colon tumour model: an investigation using dynamic contrast-enhanced MRI and the rapid clearance blood pool contrast agent, P792 (gadomelitol). NMR Biomed.

[17] Eliceiri BP, Cheresh DA (2000). Role of alpha v integrins during angiogenesis. Cancer J.

[18] Ruegg C, Alghisi GC (2010). Vascular integrins: therapeutic and imaging targets of tumor angiogenesis. Recent Results Cancer Res.

[19] Desgrosellier JS, Cheresh DA (2010). Integrins in cancer: biological implications and therapeutic opportunities. Nat Rev Cancer.

[20] Withofs N, Signolle N, Somja J, Lovinfosse P, Nzaramba EM, Mievis F, Giacomelli F, Waltregny D, Cataldo D, Gambhir SS, Hustinx R (2015). 18F-FPRGD2 PET/CT imaging of integrin alphavbeta3 in renal carcinomas: correlation with histopathology. J Nucl Med.

[21] Kenny LM, Coombes RC, Oulie I, Contractor KB, Miller M, Spinks TJ, McParland B, Cohen PS, Hui AM, Palmieri C, Osman S, Glaser M, Turton D (2008). Phase I trial of the positron-emitting Arg-Gly-Asp (RGD) peptide radioligand ^18^F-AH111585 in breast cancer patients. J Nucl Med.

[22] Haubner R, Weber WA, Beer AJ, Vabuliene E, Reim D, Sarbia M, Becker KF, Goebel M, Hein R, Wester HJ, Kessler H, Schwaiger M (2005). Noninvasive visualization of the activated alphavbeta3 integrin in cancer patients by positron emission tomography and [18F]Galacto-RGD. PLoS Med.

[23] Gaertner FC, Kessler H, Wester HJ, Schwaiger M, Beer AJ (2012). Radiolabelled RGD peptides for imaging and therapy. Eur J Nucl Med Mol Imaging.

[24] Cai W, Niu G, Chen X (2008). Imaging of integrins as biomarkers for tumor angiogenesis. Curr Pharm Des.

[25] Yang M, Gao H, Yan Y, Sun X, Chen K, Quan Q, Lang L, Kiesewetter D, Niu G, Chen X (2011). PET imaging of early response to the tyrosine kinase inhibitor ZD4190. Eur J Nucl Med Mol Imaging.

[26] Battle MR, Goggi JL, Allen L, Barnett J, Morrison MS (2011). Monitoring tumor response to antiangiogenic sunitinib therapy with ^18^F-fluciclatide, an ^18^F-labeled alphaVbeta3-integrin and alphaV beta5-integrin imaging agent. J Nucl Med.

[27] Ling Y, Yang Y, Lu N, You QD, Wang S, Gao Y, Chen Y, Guo QL (2007). Endostar, a novel recombinant human endostatin, exerts antiangiogenic effect via blocking VEGF-induced tyrosine phosphorylation of KDR/Flk-1 of endothelial cells. Biochem Biophys Res Commun.

[28] Wen QL, Meng MB, Yang B, Tu LL, Jia L, Zhou L, Xu Y, Lu Y (2009). Endostar, a recombined humanized endostatin, enhances the radioresponse for human nasopharyngeal carcinoma and human lung adenocarcinoma xenografts in mice. Cancer Sci.

[29] Ge W, Cao DD, Wang HM, Jie FF, Zheng YF, Chen Y (2011). Endostar combined with chemotherapy versus chemotherapy alone for advanced NSCLCs: a meta-analysis. Asian Pac J Cancer Prev.

[30] Han B, Xiu Q, Wang H, Shen J, Gu A, Luo Y, Bai C, Guo S, Liu W, Zhuang Z, Zhang Y, Zhao Y, Jiang L (2011). A multicenter, randomized, double-blind, placebo-controlled study to evaluate the efficacy of paclitaxel-carboplatin alone or with endostar for advanced non-small cell lung cancer. J Thorac Oncol.

[31] Ke QH, Zhou SQ, Huang M, Lei Y, Du W, Yang JY (2012). Early efficacy of Endostar combined with chemoradiotherapy for advanced cervical cancers. Asian Pac J Cancer Prev.

[32] Guan Y, Li A, Xiao W, Liu S, Chen B, Lu T, Zhao C, Han F (2015). The efficacy and safety of Endostar combined with chemoradiotherapy for patients with advanced, locally recurrent nasopharyngeal carcinoma. Oncotarget.

[33] Sun X, Yan Y, Liu S, Cao Q, Yang M, Neamati N, Shen B, Niu G, Chen X (2011). 18F-FPPRGD2 and 18F-FDG PET of response to Abraxane therapy. J Nucl Med.

[34] Beer AJ, Grosu AL, Carlsen J, Kolk A, Sarbia M, Stangier I, Watzlowik P, Wester HJ, Haubner R, Schwaiger M (2007). [18F]galacto-RGD positron emission tomography for imaging of alphavbeta3 expression on the neovasculature in patients with squamous cell carcinoma of the head and neck. Clin Cancer Res.

[35] Lee TS, Ahn SH, Moon BS, Chun KS, Kang JH, Cheon GJ, Choi CW, Lim SM (2009). Comparison of 18F-FDG, 18F-FET and 18F-FLT for differentiation between tumor and inflammation in rats. Nucl Med Biol.

[36] Strauss LG (1996). Fluorine-18 deoxyglucose and false-positive results: a major problem in the diagnostics of oncological patients. Eur J Nucl Med.

[37] Mittra ES, Goris ML, Iagaru AH, Kardan A, Burton L, Berganos R, Chang E, Liu S, Shen B, Chin FT, Chen X, Gambhir SS (2011). Pilot pharmacokinetic and dosimetric studies of (18)F-FPPRGD2: a PET radiopharmaceutical agent for imaging alpha(v)beta(3) integrin levels. Radiology.

[38] Hernandez R, Czerwinski A, Chakravarty R, Graves SA, Yang Y, England CG, Nickles RJ, Valenzuela F, Cai W (2015). Evaluation of two novel (6)(4)Cu-labeled RGD peptide radiotracers for enhanced PET imaging of tumor integrin alphavbeta(3). Eur J Nucl Med Mol Imaging.

[39] Liu S, Liu Z, Chen K, Yan Y, Watzlowik P, Wester HJ, Chin FT, Chen X (2010). 18F-labeled galacto and PEGylated RGD dimers for PET imaging of alphavbeta3 integrin expression. Mol Imaging Biol.

[40] McBride WJ, D'souza CA, Sharkey RM, Karacay H, Rossi EA, Chang CH, Goldenberg DM (2010). Improved 18F labeling of peptides with a fluoride-aluminum-chelate complex. Bioconjug Chem.

[41] Jacobson O, Zhu L, Ma Y, Weiss ID, Sun X, Niu G, Kiesewetter DO, Chen X (2011). Rapid and simple one-step F-18 labeling of peptides. Bioconjug Chem.

[42] Morrison MS, Ricketts SA, Barnett J, Cuthbertson A, Tessier J, Wedge SR (2009). Use of a novel Arg-Gly-Asp radioligand, 18F-AH111585, to determine changes in tumor vascularity after antitumor therapy. J Nucl Med.

[43] Guo N, Lang L, Gao H, Niu G, Kiesewetter DO, Xie Q, Chen X (2012). Quantitative analysis and parametric imaging of ^18^F-labeled monomeric and dimeric RGD peptides using compartment model. Mol Imaging Biol.

[44] Guo J, Guo N, Lang L, Kiesewetter DO, Xie Q, Li Q, Eden HS, Niu G, Chen X (2014). (18)F-alfatide II and (18)F-FDG dual-tracer dynamic PET for parametric, early prediction of tumor response to therapy. J Nucl Med.

[45] Lang L, Li W, Guo N, Ma Y, Zhu L, Kiesewetter DO, Shen B, Niu G, Chen X (2011). Comparison study of [18F]FAl-NOTA-PRGD2, [18F]FPPRGD2, and [68Ga]Ga-NOTA-PRGD2 for PET imaging of U87MG tumors in mice. Bioconjug Chem.

